# An Isoform of Eukaryotic Initiation Factor 4E from *Chrysanthemum morifolium* Interacts with *Chrysanthemum Virus B* Coat Protein

**DOI:** 10.1371/journal.pone.0057229

**Published:** 2013-03-07

**Authors:** Aiping Song, Wanghuai Lou, Jiafu Jiang, Sumei Chen, Zuxia Sun, Zhiyong Guan, Weimin Fang, Nianjun Teng, Fadi Chen

**Affiliations:** College of Horticulture, Nanjing Agricultural University, Nanjing, China; University of South Florida College of Medicine, United States of America

## Abstract

**Background:**

Eukaryotic translation initiation factor 4E (eIF4E) plays an important role in plant virus infection as well as the regulation of gene translation.

**Methodology/Principal Findings:**

Here, we describe the isolation of a cDNA encoding CmeIF(iso)4E (GenBank accession no. JQ904592), an isoform of eIF4E from chrysanthemum, using RACE PCR. We used the CmeIF(iso)4E cDNA for expression profiling and to analyze the interaction between CmeIF(iso)4E and the *Chrysanthemum virus B* coat protein (CVBCP). Multiple sequence alignment and phylogenetic tree analysis showed that the sequence similarity of CmeIF(iso)4E with other reported plant eIF(iso)4E sequences varied between 69.12% and 89.18%, indicating that CmeIF(iso)4E belongs to the eIF(iso)4E subfamily of the eIF4E family. CmeIF(iso)4E was present in all chrysanthemum organs, but was particularly abundant in the roots and flowers. Confocal microscopy showed that a transiently transfected CmeIF(iso)4E-GFP fusion protein distributed throughout the whole cell in onion epidermis cells. A yeast two hybrid assay showed CVBCP interacted with CmeIF(iso)4E but not with CmeIF4E. BiFC assay further demonstrated the interaction between CmeIF(iso)4E and CVBCP. Luminescence assay showed that CVBCP increased the RLU of Luc-CVB, suggesting CVBCP might participate in the translation of viral proteins.

**Conclusions/Significance:**

These results inferred that CmeIF(iso)4E as the cap-binding subunit eIF(iso)4F may be involved in *Chrysanthemum Virus B* infection in chrysanthemum through its interaction with CVBCP in spatial.

## Introduction

Eukaryotic translation initiation factor 4E (eIF4E) is a protein that plays a major role in the regulation of gene expression at the post-transcriptional level [1]. Biochemically, eIF4E functions at the levels of cap-dependent translation and nuclear mRNA export, both of which require its m^7^G cap-binding activity. eIF4E is therefore known as a cap-binding protein [2]. In translation, the initiation step is by far the most complex phase and at least 12 eukaryotic initiation factors are involved [3]. In the first step, eIF4E binds to the mRNA 5′ m^7^G cap structure and brings the mRNA into a complex with other protein synthesis initiation factors and ribosomes, resulting in mRNA circularization and translation initiation [4], [5]. eIF4E is a component of the heterotrimeric complex, eIF4F, which also includes the RNA helicase, eIF4A, and the large scaffolding protein, eIF4G [6]. It is becoming clear that most eukaryotic organisms encode and express multiple eIF4E family members, some for general translation and others for specific functions, including the control of translation [7]. The eIF4E family can vary significantly between different taxonomic groups; it is divided into two categories, eIF4E and its isoform eIF(iso)4E, in plants. Accordingly, isoforms of each component, eIF(iso)4F and eIF(iso)4G, also exist in plants [8], [9]. The eIF4E and eIF(iso)4E proteins are mechanistically equivalent during the process of translation, but exhibit differences in their expression in different tissues and their ability to bind to m^7^G and other cap analogues [10], [11]. Whereas eIF4E is present in all tissues except the root specialization zones, eIF(iso)4E is particularly abundant in floral tissues and young tissues [11]. Differences in their transcription patterns and binding affinities suggest that these isoforms might have complementary biological roles [12].

In addition to this traditional function, many studies have demonstrated that eIF4E and its isoform, eIF(iso)4E, often participate in the virus infection of plants [13]. Viruses target translation initiation factors to take over the protein synthesis machinery of the infected cells [14]. The first evidence that plant viruses interacted directly with eIF4E in plants was provided when it was shown that VPg linked to 5′ terminus of the viral RNA from *Turnip mosaic virus* (TuMV, genus *Potyvirus*) bound to *A. thaliana* eIF(iso)4E in yeast two-hybrid binding assays [15]. These findings suggest that VPg performs the functions of the cap structure of mRNA. VPg has been shown to bind to eIF4E/eIF(iso)4E proteins in several plant potyvirus systems, including lettuce, pea, wheat, melon, rice, pepper, Arabidopsis, and *Brassica rapa* [10], [12], [16]–[22]. Besides *Potyvirus*, members of certain other virus groups such as *Cucumovirus* and *Bymovirus* also interact with eIF4E/eIF(iso)4E [23], [24]. Evidence has shown that the barley *rym4* gene locus, which controls immunity to viruses in the genus *Bymovirus*, corresponds to eIF4E [25]. *CUM1* encodes eIF4E protein, the *cum1* mutation of *Arabidopsis thaliana* inhibits cucumber mosaic virus (CMV) multiplication by decreasing the accumulation of CMV 3a proteins, which is necessary for cell-to-cell movement of the virus [24]. Therefore, eIF4E/eIF(iso)4E may not only participate in the infection and translation of viruses, but it may also affect virus movement.

The eIF4E and eIF(iso)4E proteins exhibit differences in their ability to bind m^7^G and other cap analogues. Hence, viruses selectively combine with either eIF4E or eIF(iso)4E. When mutated *Arabidopsis thaliana* eIF4E and eIF(iso)4E were tested for susceptibility to *Clover yellow vein virus* (ClYVV) and *Turnip mosaic virus* (TuMV), eIF4E was shown to be necessary for infection by ClYVV but not for TuMV, while eIF(iso)4E was in contrast [26]. Different viruses infect the same plants, and the same virus infects different plants, by interaction with different members of the eIF4E family. For example, *Lettuce mosaic virus* (LMV) and *Tobacco etch virus* (TEV) infect *Arabidopsis* by interacting with eIF(iso)4E [27], [28], but infect pepper, tomato, and lettuce by interacting with eIF4E [12], [13], [29].

The mRNAs of many plant RNA viruses lack a cap structure, a poly (A) tail or both, yet they efficiently compete with host mRNAs for the translational machinery. Several host proteins that interact with viral coat proteins are involved in the cell-to-cell movement or subcellular localization of viruses [30]–[32]. Furthermore, a report suggested that translation of *Alfalfa mosaic virus* (AMV) genomic RNA is enhanced by binding of several coat protein molecules to its 3′ end, apparently mimicking the function of poly(A)-binding protein (PABP) [33]. GST pull-down revealed that AMV coat protein interacts with eIF4F and eIF(iso)4F from wheat germ [34]. In the genera *Alfamovirus* and *Ilarvirus*, initiation of infection by viruses requires the addition of coat protein (CP) to a mixture of the genomic RNAs [35]–[37]. Chrysanthemum is ranked among the top ten most important flower crops in the international cut-flower market [38]. Several viruses and viroids have been reported in chrysanthemum, and *Chrysanthemum virus B* (CVB, genus *Carlavirus*, family *Flexiviridae*) is one of the major pathogens of chrysanthemum [39]. The *Carlavirus* genome is a positive, single-strand RNA with a 5′-cap and 3′-poly(A) structure that contains six open reading frames (ORFs), of which ORF5 encodes the coat protein [40]. In this study, we report the cloning of a full-length cDNA encoding eIF(iso)4E from chrysanthemum. We used the cloned cDNA for expression profiling of the *CmeIF(iso)4E* gene. Finally, we demonstrated the interaction of CVBCP with CmeIF(iso)4E by yeast two-hybrid assay and BiFC (Bimolecular Fluorescence Complementation) in onion epidermal cells, and that CVBCP increased the RLU of Luc-CVB through Luminescence assay, suggesting CVBCP might participate in the translation of viral proteins.

## Materials and Methods

### Plants Materials

The chrysanthemum variety ‘Jinba’ and diseased leaves infected with CVB were obtained from the Chrysanthemum Germplasm Resource Preserving Centre, Nanjing Agricultural University, China. *Arabidopsis thaliana* Columbia-0 plants were grown in 8 h photoperiod at 23 °C in 50–60% humidity.

### *CmeIF(iso)4E* Full-Length cDNA Cloning and Sequence Analysis

Total RNA was isolated from chrysanthemum ‘Jinba’ leaves using the RNAiso reagent (Takara, Japan) according to the manufacturer’s instructions. The cDNA first strand was synthesized from 1 µg of total RNA using the M-MLV RTase cDNA Synthesis kit (Takara, Japan) according to the manufacturer’s instructions. A gene-specific primer pair (i4E-F/-R) was designed to amplify a fragment of *CmeIF(iso)4E* based on the sequences from other plants, and RACE PCR was then used to obtain the full-length cDNA. For the 3’ RACE reaction, the first strand cDNA was synthesized using an d(T)-adapter primer incorporating the sequence of the adaptor primer, and this was followed by a nested PCR using primer pair i4E-3′GSP 1/2/3 and the adaptor primer (Table 1). For the 5’ RACE, the nested PCR was performed using primers AAP and AUAP provided by the 5’ RACE System kit v2.0 (Invitrogen), along with gene-specific primer i4E-5′GSP1/2/3 (Table 1). PCR products were purified using a Biospin Gel Extraction kit (BioFlux, China) and cloned into the pMD19-T easy vector (Takara) for sequencing. Finally, a pair of gene-specific primers (eIF(iso)4E-F and eIF(iso)4E-R) was designed from the putative 5′ and 3′ UTR sequences to amplify the complete *CmeIF(iso)4E* open reading frame (ORF). The sequences of all the above primers are given in Table 1. The CmeIF(iso)4E amino acid sequence was aligned with the sequences of its homologues using DNAMAN software. The phylogenetic tree file was produced using ClustalW (http://www.ebi.ac.uk/clustalW/) [41].

**Table 1:** Names and sequences of primers used in this study.

Primer name	Sequence (5′–3′)	
i4E-F	5′ CACCTTCGACACCGTGGARGANTTYTGG 3′	
i4E-R	5′ CGTCGGCCTCGTCGAAYTGYTCNCC 3′	
d(T)-adapter	5′ AAGCAGTGGTATCAACGCAGAGTAC(T)_15_ 3′	
adapter	5′ AAGCAGTGGTATCAACGCAGAGTAC 3′	
i4E-3′GSP1	5′ TGGAAGAGTTCTGGTGTTTGTATG 3′	
i4E-3′GSP2	5′ GGATTGAGCCTAAATGGGAAGA 3′	
i4E-3′GSP3	5′ AGAAAGGCTGGACTTGAGACTATG 3′	
AAP	5′ GGCCACGCGTCGACTAGTACGGGIIGGGIIGGGIIG 3′	
AUAP	5′ GGCCACGCGTCGACTAGTAC 3′	
i4E-5′GSP1	5′ TGACCTTGTCTTAGAATCATCGTG 3′	
i4E-5′GSP2	5′ CGTCAGCAGCATTCTTAGTCCAT 3′	
i4E-5′GSP3	5′ TAGCAACCACGCCACAGATT 3′	
eIF(iso)4E-F	5′ CCTGAAAAAAAATCATGGCGGCA 3′	
eIF(iso)4E-R	5′ ATTCTGTTATGCCCAGGAGT 3′	
CVBCP-F	5′ ATGCCTCCCAAACCGGC 3′	
CVBCP-R	5′ TTATAATGTCTTATTATTCGCATTG 3′	
i4E-RT-F	5′ ATGGACTAAGAATGCTGCTAACGAG 3′	
i4E-RT-R	5′ TTATGCCCAGGAGTCACACGCTATA 3′	
GAPDH-F	5′ GCTGTATCCCCATTCGTT 3′	
GAPDH-R	5′ AGAAGGCAAGCTCAAGGG 3′	
i4E-SL -F	5′ GCGTCGACATGGCGGCGAATGATGGC 3′	(*Sal*I)
i4E-SL -R	5′ TTGCGGCCGCGACACGCTATATCGACCCTTTG 3′	(*Not*I)
AD-4E-F	5′ CCGGAATTCATGGTTGAAGAGCACCACACC 3′	(*Eco*RI)
AD-4E-R	5′ CGCGGATCCCTGCTGAATATTTGTTTTTGGCATT 3′	(*Bam*HI)
AD-i4E-F	5′ CCGGAATTCATGGCGGCGAATGATGGC 3′	(*Eco*RI)
AD-i4E-R	5′ CGCGGATCCCCACGCTATATCGACCCTTTG 3′	(*Bam*HI)
BD-CVBCP-F	5′ CGGAATTCATGCCTCCCAAACCGGC 3′	(*Eco*RI)
BD-CVBCP-R	5′ ACGCGTCGACGTTATAATGTCTTATTATTCGCATTG 3′	(*Sal*I)
BiFC-i4E-F	5′ CCGGAATTC ATGGCGGCGAATGATGGC 3′	(*Eco*RI)
BiFC-i4E-R	5′ CGCGGATCCCCACGCTATATCGACCCTTTG 3′	(*Bam*HI)
BiFC-CVBCP-F	5′ CGGAATTCATGCCTCCCAAACCGGC 3′	(*Eco*RI)
BiFC-CVBCP-R	5′ CCCCCGGGTTATAATGTCTTATTATTCGCATTG 3′	(*Sma*I)
CVB-3UTR-R1	5′ GCAGCTACTACTGAGCTCGAATTCC 3′	
CVB-3UTR-R2	5′ TATATTAATTAGGCTTTAGAAGCAGCTACTACTG 3′	
CVB-3UTR-R3	5′ CGGGGTACCTTATAGTTTCACACCTTATATATTAATTAGGC	(*Kpn*I)
Luc-Nco-F	5′ GTCGACCATGGAAGACGCCA 3′	(*Nco*I)

Note: underlined sequences indicate restriction enzyme sites, the names of which are shown in brackets.

### CVB CP cDNA Cloning and Sequencing

Total RNA was isolated from infected leaves using the RNAiso reagent (Takara) according to the manufacturer’s instructions. The cDNA first strand was synthesized from 1 µg of total RNA using the M-MLV RTase cDNA Synthesis kit (Takara) and a random primer incorporating the sequence of the adaptor primer according to the manufacturer’s instructions. According to the reported cDNA sequence of the CVB coat protein (GenBank: AJ879077.1), we designed a pair of gene-specific primers (CVBCP-F and CVBCP-R) to amplify the complete *CVBCP* open reading frame (ORF). PCR products were purified using a Biospin Gel Extraction kit (BioFlux) and cloned into the pMD19-T easy vector (Takara) for sequencing.

### Quantitative Real-Time PCR (qRT-PCR)

To determine the expression pattern of *CmeIF(iso)4E* in different tissues in chrysanthemums, total RNA was extracted from roots, stems, leafs, tubular flowers, and ligulate flowers of chrysanthemum ‘Jinba’ using the RNAiso reagent (Takara), and potential contaminating genomic DNA was removed by DNase I treatment. cDNA was synthesized using the M-MLV RTase cDNA Synthesis kit (Takara), and quantitative real-time PCR (qRT-PCR) was performed using SYBR_Green I (TOYOBO, Japan) in a Rotor-Gene 3000 (Corbett, Australia). The primer pair i4E-RT-F/-R (Table 1) was used to amplify a 158 bp fragment in the 3′ region of the gene, avoiding the more well-conserved segments of the gene. The chrysanthemum *CmGAPDH* gene (GenBank accession number: DK941612) was used as a reference. Each 25 µl qRT-PCR reaction contained 10 µl SYBR Green PCR master mix, 0.2 µM of each primer and 10 ng cDNA, and the amplification regime consisted of an initial denaturation of 95°C for 60 s, followed by 40 cycles of 95°C for 15 s, 60°C for 15 s, and 72°C for 45 s. The resulting data were represented as the means ± SD of three replicates. Relative expression levels were calculated by the 2^-ΔΔCT^ method, where ΔCT  =  (CT, Target - CT, GAPDH), and the PCR signals were normalized to that of root.

### Subcellular Localization of CmeIF(iso)4E

The plasmid used for transient transfection was generated using the Invitrogen Gateway system according to the manufacturer’s instructions. The *CmeIF(iso)4E* ORF, lacking its stop codon, was amplified using a Phusion^®^ High-Fidelity PCR kit (New England Biolabs, USA) with the primer pair, i4E-SL-F/-R, then subcloned into the pMD19-T vector (Takara) and confirmed by DNA sequencing. The confirmed gene from the pMD19-T vector was inserted into the pENTR^TM^ 1A dual selection vector (Invitrogen, USA) at *Sal* I and *Not* I with T4 DNA ligase (Fermentas, Canada), and then the construct was recombined with pEarleyGate 103 [42] to construct a CmeIF(iso)4E-GFP fusion vector, using the LR Clonase^TM^ II enzyme mix (Invitrogen). Plasmid DNA was transiently introduced into onion (*Allium cepa*) epidermal cells using a helium-driven particle accelerator (PDS-1000; Bio-Rad, USA) according to the manufacturer’s instructions. After bombardment, the onion peels were incubated for 16 h on Murashige and Skoog plates in the dark. Confocal laser microscopy (Leica SP2, Germany) was used to monitor the expression of GFP.

### Yeast Two-Hybrid Assay

The yeast two-hybrid assay was performed with the Matchmaker™ Gold Yeast Two-Hybrid System (Clontech, USA). The coding region of *CmeIF(iso)4E* amplified with the primer pair, AD-i4E-F/-R, was inserted into the pGADT7 prey vector at the *Eco*RI / *Bam*HI sites (fused to the GAL4 activation domain) to produce pAD-CmeIF(iso)4E. The CVB CP gene was amplified using the primer pair, BD-CVBCP-F/-R, and cloned into the yeast bait expression vector, pGBKT7, at the *EcoR*I / *Sal* I sites (fused to the Gal4 DNA binding domain) to produce pBD-CVBCP. The interactions between CmeIF(iso)4E and CVBCP were identified by testing combinations of CVBCP-bait and CmeIF(iso)4E-prey constructs co-expressed in the yeast Y2HGold strain, according to the manufacturer’s instructions. Interactions were detected by growth on the selective media, SD/-Trp/-Leu and SD/-Trp/-Leu/-His/-Ade, according to the manufacturer’s instructions. Yeast diploids containing empty pGBKT7 and pGADT7 were used as negative controls. α-Galactosidase activity was assayed on filters as described in the Yeast Protocol Handbook (Clontech). All primers used in the yeast two-hybrid assays are listed in Table 1.

### Bimolecular Fluorescence Complementation Assay

In the BiFC constructs, the coding region of *CmeIF(iso)4E* was cloned as N-terminal fusions to the fluorescent protein fragments in the pSAT4A-nEYFP-N1 vector at the *Eco*RI / *Bam*HI cloning sites using the primer pair, BiFC-i4E-F/-R. The full-length cDNA encoding the coat protein of CVB was cloned as C-terminal fusions to the fluorescent protein fragments in the pSAT4A-cEYFP-N1 vector at the *Eco*RI / *Sma*I cloning sites using the primer pair, BiFC-CVBCP-F/-R. Transient gene expression in onion epidermal cells was performed using a Biolistic PDS-1000/He Particle Delivery System (Bio-Rad) according to the manufacturer’s instructions. After bombardment, the onion peels were incubated for 16 h on Murashige and Skoog plates in the dark. YFP fluorescence images were acquired by confocal laser scanning microscopy (model TCS SP2; Leica). All primers used in bimolecular fluorescence complementation assays are listed in Table 1.

### Luminescence assay

The Luc-CVB vector was constructed via adding the 3’UTR of CVB between the luciferase ORF and 35S terminator in the pCAMBIA99-1-Luc plasmid. The detailed construction procedures are described below. Triple nested PCR was amplified using a Phusion® High-Fidelity PCR kit (New England Biolabs, USA) with the primer pairs (1^st^ Luc-Nco-F/ CVB-3UTR-R1, 2^nd^ Luc-Nco-F/ CVB-3UTR-R2, 3^rd^ Luc-Nco-F/ CVB-3UTR-R3). The PCR products were purified using a Biospin Gel Extraction kit (BioFlux). The purified Luc-CVB fragment and pCAMBIA99-1-Luc plasmid were digested by *Nco*I/ *Kpn*I, then the digested Luc-CVB fragment was inserted into digested pCAMBIA99-1-Luc to replace Luc with Luc-CVB. The generated Luc-CVB vector was confirmed by sequencing.

Protoplasts were prepared and transfected based on the protocol described by Yoo et al. [43]. For Luc (pCAMBIA99-1-Luc), Luc-CVB (pCAMBIA99-1-Luc-CVB) and CVBCP (pSAT4A-CVBCP) transfection, 7.5 µg of pCAMBIA99-1-Luc, pCAMBIA99-1-Luc-CVB and pSAT4A-CVBCP plasmid was transfected respectively; while for Luc+CVBCP and Luc-CVB+CVBCP transfection, additional 7.5 µg pSAT4A-CVBCP plasmid was added. ddH_2_O was transfected as a mock.

The luciferase activities were measured 16 h after the transfection. Luminescence was detected according the protocol described by Fujikawa and Kato with minor modifications [44]. The beetle luciferin (Promega, Madison, Wisconsin, USA) was dissolved in sterile water to a final concentration of 7.8 mM and stored at -80°C. The stock solutions were diluted 10 times with the W5 buffer before each assay. 10 µl of diluted beetle luciferin was added to each well of a 96-well plate (PerkinElmer, Waltham, Massachusetts, USA), then added 150 µl transfected protoplasts, mixed well. After 15 min of incubation at room temperature in the dark, the luminescence in each well was quantified with 10 sec integration periods using a Glomax 96 microplate luminometer (Promega). Each assay repeated three times.

## Results

### Identification and characterization of CmeIF(iso)4E, an isoform of translation initiation factor 4E

**Figure 1:**
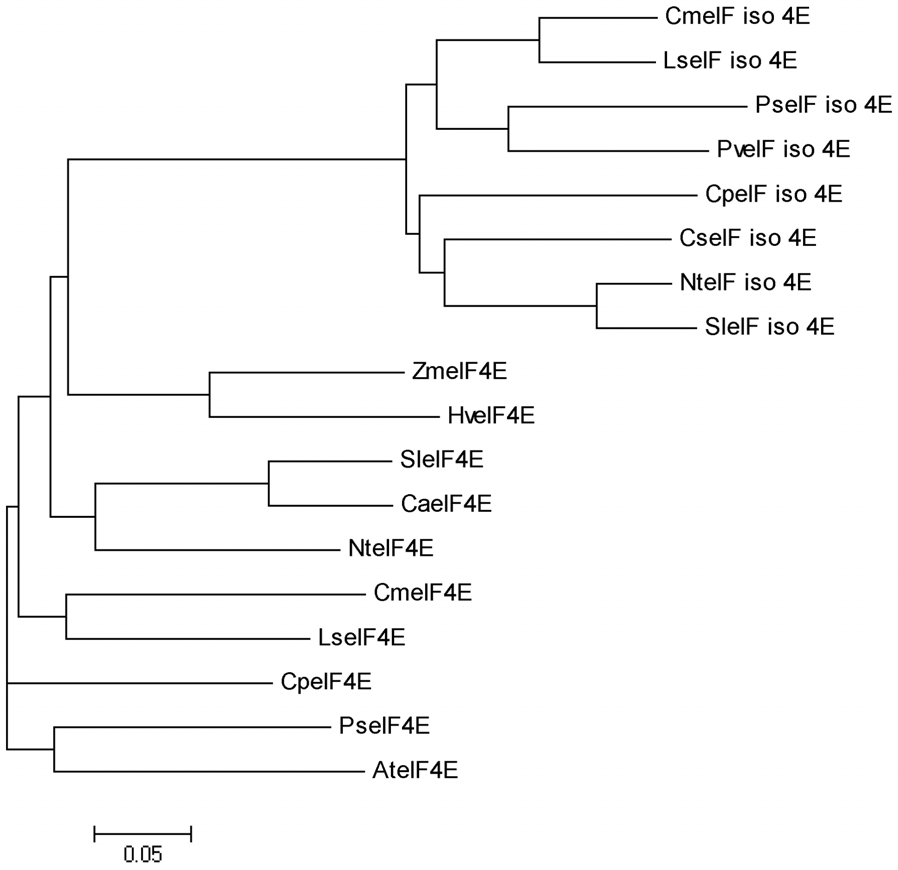
Phylogenetic relationship between CmeIF(iso)4E and other eIF4E superfamily proteins. The phylogenetic tree file was produced using ClustalW (http://www.ebi.ac.uk/clustalW/). The GenBank accession numbers of the amino acid sequences used are: CmeIF_iso_4E (JQ904591, *Chrysanthemum morifolium*), LseIF_iso_4E (AAP86603.1, *Lactuca sativa*), SleIF_iso_4E (NP_001234772.1, *Solanum lycopersicum*), PseIF_iso_4E (BAK53449.1, *Pisum sativum*), NteIF_iso_4E (AAU06579.1, *Nicotiana tabacum*), PveIF_iso_4E (ABU54807.1, *Phaseolus vulgaris*), CpeIF_iso_4E (ACM18197.1, *Carica papaya*), CseIF_iso_4E (ABY56102.1, *Cucumis sativus*), CmeIF4E (JQ904591, *Chrysanthemum morifolium*), AteIF4E (NP_193538.1, *Arabidopsis thaliana*), CaeIF4E (AAN74644.1, *Cayenne pepper*), CpeIF4E (ACN38307.1, *Carica papaya*), HveIF4E (CAR92170.2, *Hordeum vulgare*), LseIF4E (AAP86602.1, *Lactuca sativa*), NteIF4E (DK22107.1, *Nicotiana tabacum*), SleIF4E (AAV88610.1, *Solanum lycopersicum*), PseIF4E (ABG35119.1, *Pisum sativum*) and ZmeIF4E (ACG34414.1, *Zea mays*).

Based on the amino acid sequences of eIF4E and eIF(iso)4E proteins from other plants, a eIF4E homologous gene was isolated by RT-PCR and RACE that was designated as *CmeIF(iso)4E* (GeneBank accession JQ904592). The cloned *CmeIF(iso)4E* cDNA consists of 818 bp with a 573 bp ORF encoding a 190-amino acid protein. A homology blast showed that CmeIF(iso)4E is substantially homologous to other eIF(iso)4E proteins from other species, with sequence identity between 69.12% and 89.18%, and highest similarity to CmeIF(iso)4E from *Lactuca sativa*. Phylogenetic analysis showed that CmeIF(iso)4E is clustered with the eIF(iso)4E subgroup and separated from the eIF4E subgroup (Fig. 1).

### Expression profiling of *CmeIF(iso)4E*

**Figure 2:**
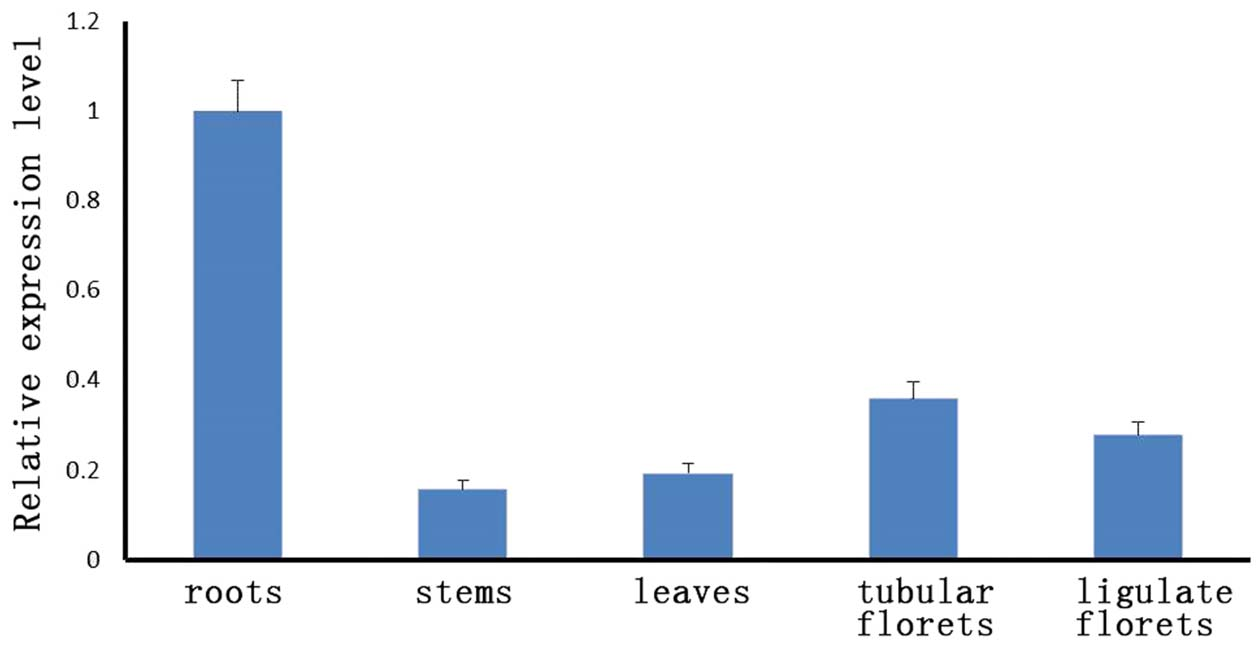
*CmeIF(iso)4E* expression in chrysanthemum as demonstrated by qRT-PCR.

**Figure 3:**
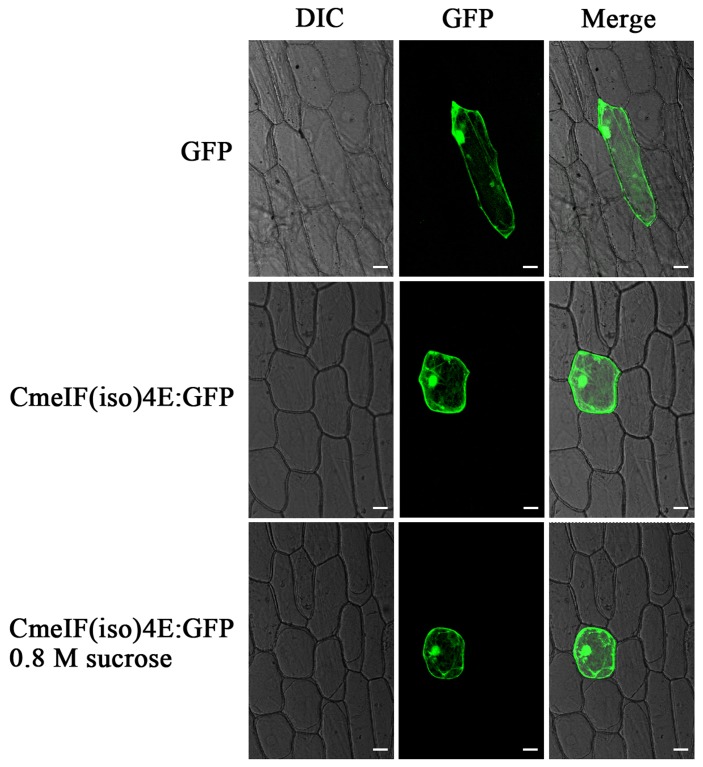
Subcellular localization of the CmeIF(iso)4E protein in onion epidermis cells. The upper row shows the 35S::GFP signal alone as a positive control; the middle row displays the signal from 35S::CmeIF(iso)4E-GFP; the lower row shows 35S::CmeIF(iso)4E-GFP following treatment with 0.8 M sucrose to induce plasmolysis. The left panel shows bright field images; the middle panel shows green fluorescence signals detected at 488 nm; the right panel shows the merged GFP signals and bright field images. Bars = 50 µm

The results of qRT-PCR showed that *CmeIF(iso)4E* is transcribed in the roots, stems, leaves, tubular florets, and ligulate florets, with the highest level of transcription being present in the root and the lowest in the stem (Fig. 2). To determine the distribution of CmeIF(iso)4E in cells, we used a transient assay involving the bombardment of CmeIF(iso)4E-GFP fusion constructs into onion (*Allium cepa*) epidermal cells. The results demonstrated that the CmeIF(iso)4E-GFP fusion protein was present in the nucleus, cytoplasm, and cytomembrane (Fig. 3).

### CmeIF(iso)4E interacts with CVBCP

**Figure 4:**
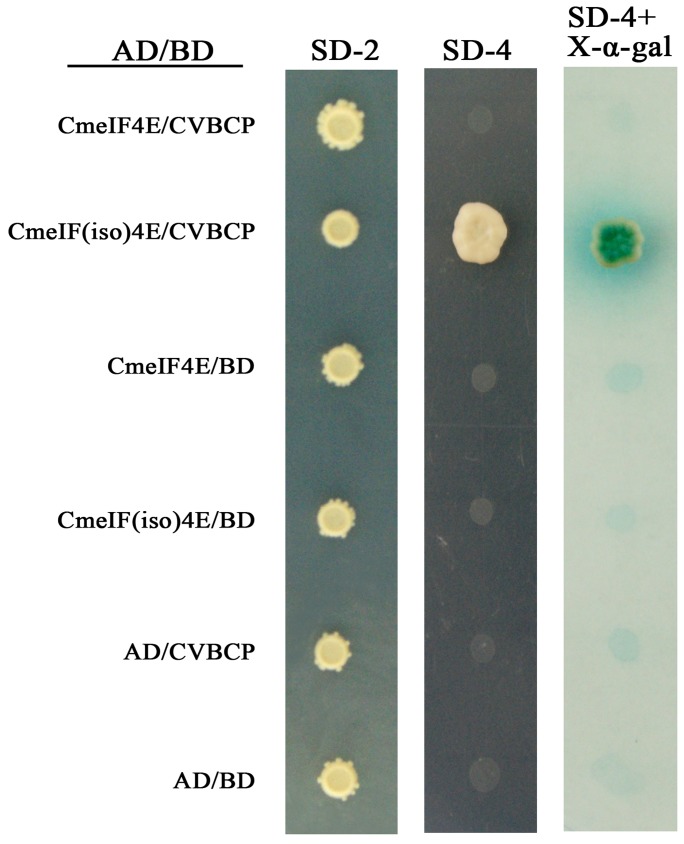
Interaction between CmeIF(iso)4E and CVBCP *in vitro* as demonstrated by Y2H assay. Yeast two-hybrid screen demonstrating the interaction between CVBCP and CmeIF(iso)4E. The left panel shows the growth of yeast cells containing both plasmids on SD-Leu-Trp medium (SD-2); the middle panel shows the selection of yeast colonies on SD/-Leu/-Trp/-Ade/-His medium (SD-4); the right panel shows the selection of yeast colonies on SD-4 containing α- X-Gal.

**Figure 5:**
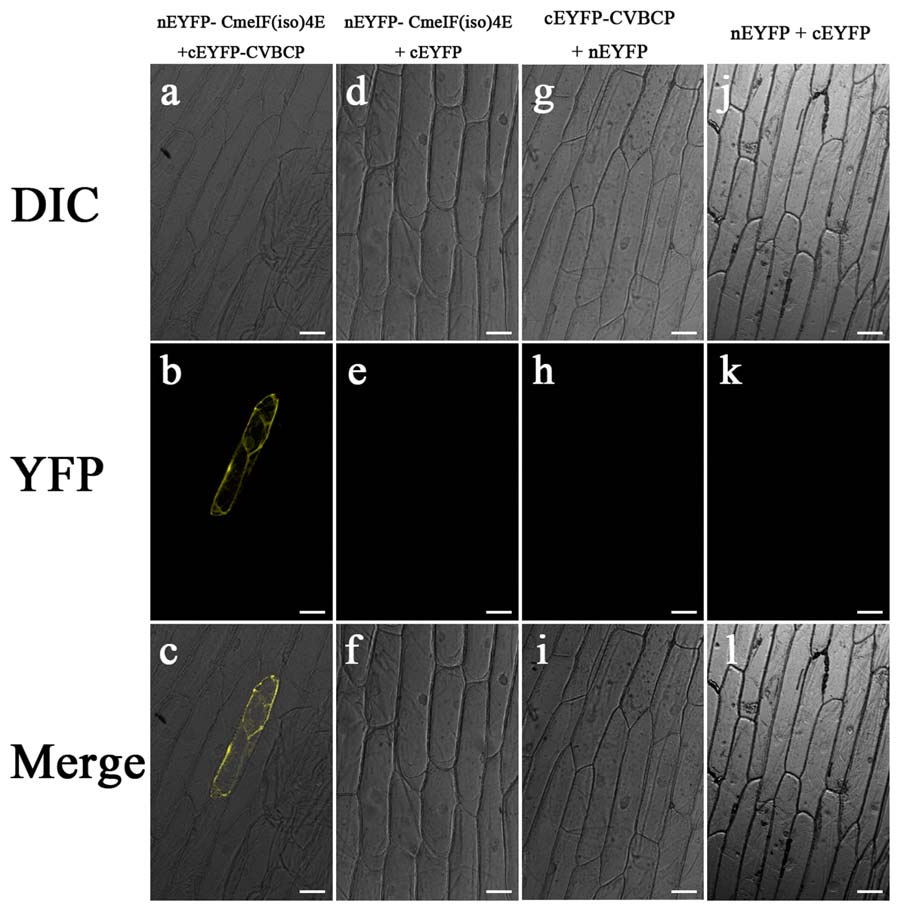
BiFC view of the interaction between CmeIF(iso)4E and CVBCP in transiently transfected onion cells. Confocal microscopy images showing yellow fluorescence in onion cells transfected with nEYFP-CmeIF(iso)4E and cEYFP-CVBCP. No fluorescence was observed in negative control onion cells co-transfected with nEYFP-CmeIF(iso)4E + cEYFP, nEYFP + cEYFP-CVBCP, or nEYFP + cEYFP. The corresponding differential interference contrast (DIC) images are shown at the top. Bars = 50 µm.

Yeast two-hybrid heterologous expression was used to assess the possible interaction between CVBCP and CmeIF(iso)4E. The CVB *CP* gene was fused to the DNA-binding domain in the bait plasmid, pGBKT7, and the *CmeIF(iso)4E* gene was cloned into the prey plasmid, pGADT7. In addition, empty vectors were transformed as negative controls for each recombinant plasmid. Screening for putative interactions was performed using SD/-Trp/-Leu/-His/-Ade media, as shown in the middle panel of Fig. 4. Fig. 4 shows that CmeIF(iso)4E grew significantly when co-transformed with CVBCP as bait, which was confirmed by positive α-X-galactosidase assays. By contrast, pGADT7 co-transformed with CVBCP or pGBKT7 as bait failed to induce growth on SD-4 media or show α-X-galactosidase activity. The interaction of CVBCP and CmeIF(iso)4E in yeast was further confirmed by BiFC. A YFP fluorescence signal was observed in the onion epidermis cells co-expressing nEYFP-CmeIF(iso)4E and cEYFP-CVBCP (Fig. 5). In contrast, no fluorescence signal was generated in cells co-expressing nEYFP-CmeIF(iso)4E and cEYFP, nEYFP and cEYFP-CVBCP, or nEYFP and cEYFP, as controls. Thus, these observations demonstrate that CmeIF(iso)4E protein interacts with CVBCP in plant cells.

### CVBCP involves in the translation process of CVB RNA

**Figure 6:**
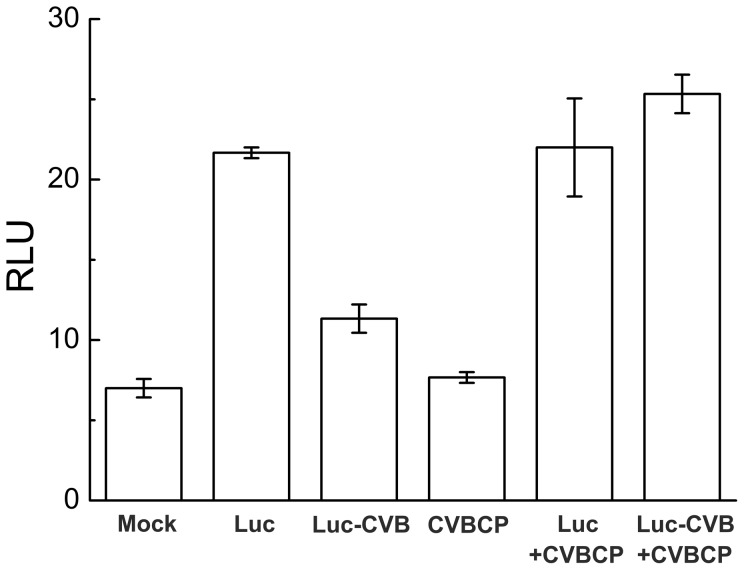
Relative luciferase activities in Arabidopsis mesophyll protoplasts after transfection with CVBCP and Luc-CVB constructs. Mock was a negative control without the constructs. Data are the mean of three independent experiments. RLU, relative light units.

The relative light units (RLU) of Luc-CVB was lower than that of Luc transfectants in Arabidopsis protoplasts, which indicated that the 3’UTR of CVB RNA might be targeted by plant immune systems (Fig. 6). The cotransfection of CVBCP and Luc-CVB recovered the activity of Luc-CVB to that of Luc (Fig. 6), which inferred that CVBCP involves in the translation process of CVB RNA.

## Discussion

Previous studies have demonstrated that the cap-binding protein, eIF4E/eIF(iso)4E, is responsible for conferring resistance to RNA viruses in plant systems. In this study, we cloned *eIF(iso)4E* from chrysanthemum using degenerate primers. Protein sequence and phylogenetic analysis revealed that CmeIF(iso)4E is highly conserved with its orthologues in other plant species. The genetic relationship between related species coincides with plant taxonomy, with the closest relatives being chrysanthemum and lettuce. The sequence identity of *CmeIF(iso)4E* to other reported plant *eIF(iso)4E* genes varied between 69.12 and 89.18%. Diversity between eIF(iso)4E proteins from different organisms mainly resides in their N-termini, which may have very different lengths and seem not to be directly involved in cap-binding [45], [46]. Functional differences also exist in the promotion of viral infection and accumulation in plant hosts. qRT-PCR showed that the transcription of CmeIF(iso)4E is highest in young roots and flowers, which is consistent with previous reports showing that eIF(iso)4E is particularly abundant in floral tissues and young tissues [11]. Other studies have shown that eIF4E and eIF(iso)4E both function in the selection of mRNA for translation, but differ in their expression in different tissues and their ability to bind to mRNA cap structures [4], [11]. Confocal microscopy showed that CmeIF(iso)4E is localized in the nucleus, cytoplasm, and cytomembrane in onion epidermal cells. In animals, eIF4E is localized predominantly in the cytoplasm, with between 12% and 25% of eIF4E being found in the nucleus [2], [47], [48]. In eukaryotic cells, eIF4E functions in the selective transport of specific mRNAs from the nucleus to the cytoplasm [49]. Here, we detected CmeIF(iso)4E on the cytomembrane by confocal microscopy, and this localization was confirmed by BiFC. Further research is needed to determine the function of CmeIF(iso)4E on the cytomembrane.

It has been shown that *Potyvirus* uses eIF4E in one host and eIF(iso)4E in another host for protein translation; for example, Tobacco etch virus (TEV) uses eIF4E in pepper [9] and tomato [50], but eIF(iso)4E in *A. thaliana* [51]. Here, we report the interaction between the coat protein of CVB and eIF(iso)4E from chrysanthemum. In the yeast two-hybrid system, we found that CVBCP interacts with CmeIF(iso)4E. This interaction was confirmed by BIFC assays, in which the co-expression of CmeIF(iso)4E and CVBCP were observed by significant yellow fluorescent in the nucleus, cytoplasm, and cytomembrane. Therefore, our results suggest that eIF4E and eIF(iso)4E are selectively involved in plant-virus interactions, and the isoforms may have complementary biological roles.

In *Potyvirus*, the interaction between VPg and eIF4E may be important for the cellular transport and localization of RNA [51]. eIF4E based resistance has also been postulated to act at the level of virus cell-to-cell movement. In pea and pepper, eIF4E assists *Potyvirus* cell-to-cell movement [19]. eIF(iso)4E is also involved in resistance to other viruses, such as CMV, and resistance to *Bymovirus* has also been reported [3],[23]. The recent demonstration of the binding of eIF4E and eIF(iso)4E to helper component proteinase (HCpro) of three potyviruses (*Potato virus A*, *Potato virus Y*, and *Tobacco etch virus*), further extends this potential interaction network [52]. It has been reported that extension of the 3' termini of AMV genomic RNAs with a poly(A) tail of 40 or 80 residues permits the initiation of infection of tobacco plants and protoplasts at a level that was 5% of the CP-mediated initiation of infection [33]. Therefore, binding of CP to the 3′ termini of AMV RNAs is functionally equivalent to the binding of PABP to the poly(A) tail of cellular mRNAs [33]. The poly(A) tail of cellular messengers enhances translation synergistically with the cap structure by mediating the interaction of poly(A)-binding PABP with the cap-binding initiation factors, eIF4F and eIF4B [53]. The reported assays revealed that AMV CP interacts specifically with the eIF4G and eIF(iso)4G subunits from wheat eIF4F and eIF(iso)4F, respectively [33]. In the genera *Alfamovirus* and *Ilarvirus*, initiation of infection by viruses requires the addition of coat protein to a mixture of the genomic RNAs in multiple steps of the replication cycle [35]. CVBCP increased the RLU of Luc-CVB, suggesting CVBCP might participate in the translation of CVB viral proteins (Fig. 5, 6). The viral RNA of CVB has the 5’ cap structure and 3’ poly(A) tail [54], the virus would recruit eIF4E of host plant cell to translate the viral protein [55]. Together with the observation of interaction between CVBCP and CmeIF(iso)4E, we suppose that CmeIF(iso)4E might regulate the viral translation via CVBCP. The eukaryotic translation initiation factors are involved in virus translation, these host factors also participate in viral RNA replication [55]. Here, our results inferred that CmeIF(iso)4E as the cap-binding subunit eIF(iso)4F may be involved in Chrysanthemum Virus B infection in chrysanthemum through its interaction with CVBCP in spatial.
